# circNINL facilitates aerobic glycolysis, proliferation, invasion, and migration in lung cancer by sponging miR-3918 to mediate FGFR1 expression

**DOI:** 10.1186/s40001-024-01636-7

**Published:** 2024-01-20

**Authors:** Sai Li, Chun Qiu, DaTong Sun, ShengHui Yang, Lin Wang

**Affiliations:** Department of Medical Oncology, Hainan General Hospital (Hainan Affiliated Hospital of Hainan Medical University), No. 19, Xiuhua Road, Xiuying District, Haikou City, 570311 Hainan China

**Keywords:** Lung cancer, Circular RNA NINL, MicroRNA-3918, FGF receptor 1, Glycolysis, Carcinogenesis

## Abstract

Previously characterized as an oncogenic player in breast cancer, the function of circular RNA NINL (circNINL) in lung cancer (LC) remained elusive. This study aimed to delineate the biological role of circNINL in LC and to unveil its potential molecular mechanisms. We discovered elevated expression levels of circNINL and Fibroblast Growth Factor Receptor 1 (FGFR1) concomitant with diminished expression of microRNA-3918 (miR-3918) in LC specimens. Knockdown of circNINL led to a marked decrease in cell proliferation, migration, invasion, and aerobic glycolysis, alongside an upsurge in apoptosis in LC cells. Either downregulation of miR-3918 or overexpression of FGFR1 mitigated the suppressive impact of circNINL knockdown on LC pathogenesis. Mechanistic studies validated that circNINL served as a competitive endogenous RNA for miR-3918, thus influencing FGFR1 expression. Further, in vivo experiments using nude mouse xenograft models underscored that silencing circNINL substantially curtailed tumor growth in LC. Collectively, these findings illuminate that circNINL exacerbates LC malignancy via the miR-3918/FGFR1 axis, a process integrally linked with the activation of aerobic glycolysis.

**Graphical Abstract:**

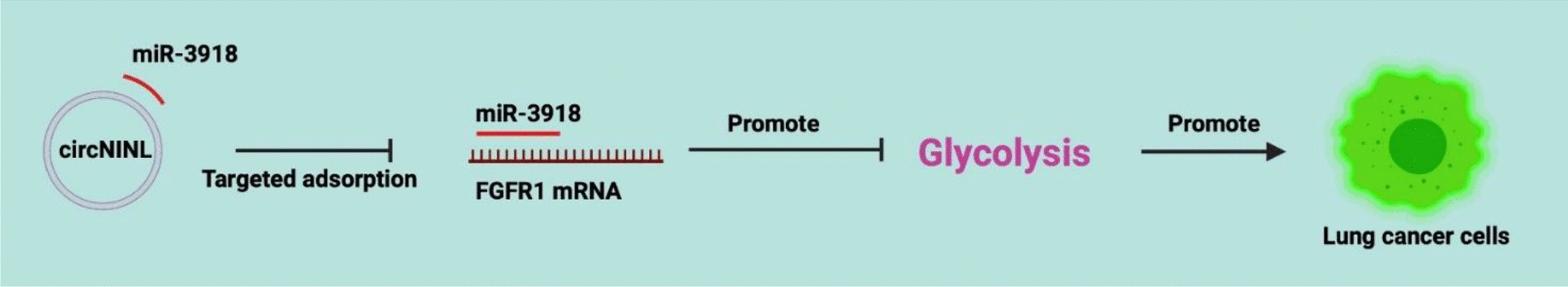

## Highlights

1. CircNINL and FGFR1 are highly expressed and miR-3918 is poorly expressed in lung cancer.
2. Knockdown of circNINL inhibits lung cancer growth and aerobic glycolysis.
3. Knockdown of miR-3918 or overexpression of FGFR1 reverses the inhibitory effect of knockdown of circNINL on lung cancer.
4. circNINL acts as a sponge for miR-3918 to mediate FGFR1 expression.

## Introduction

Taking the highest morbidity and mortality, lung cancer (LC) is extremely threatening [1] a 5-year survival rate of 4–17% [2]. At present, surgery, radiotherapy, and chemotherapy are the main traditional methods for the treatment of LC [3], but the 5-year survival rate is still less than 20% due to the high rate of metastasis and recurrence [4]. Furthermore, many patients are diagnosed at an advanced stage due to the lack of early biomarkers. Therefore, identifying effective therapeutic targets is necessary for treatment strategies for LC.

CircRNAs, a unique class of non-coding RNAs, feature a closed-loop structure, differing from traditional linear RNAs [5]. This formation endows them with notable stability and resistance to RNAse degradation. Functionally diverse, circRNAs act chiefly as microRNA 'sponges,' influencing gene regulation by modulating microRNAs (miRNAs) activity [6]. Their roles extend to protein synthesis regulation and gene transcription, underscoring their multifaceted biological significance [7]. In recent years, circRNAs, conserved and closed circular RNAs, have been widely considered to act importantly in the development of cancer, including LC [8, 9]. CircRNAs abnormally expressed in LC are involved in various biological processes of LC, such as glycolysis. It has been reported that circCPA4 promotes the growth of LC cells [10] and circ-METTL15 contributes to LC immune escape [11]. It can be seen that the regulation of circRNAs has a profound impact on the progression of LC. circNINL is a novel circRNA is up-regulated in breast cancer and promote breast cancer progression [12], but little is known about its function in LC.

The main regulatory mechanism of circRNA is to act as a sponge to adsorb miRNAs, and miRNAs target downstream mRNAs to play a biological role [2]. Therefore, based on the circRNA-miRNA-mRNA regulatory network, we predicted a miRNA (miR-3918) and a miRNA target gene (FGFR1) associated with circNINL. miR-3918 has been implicated in gastric cancer [13] and hepatocellular carcinoma [14] as a tumor suppressor. Fibroblast growth factor receptor 1 (FGFR1) functions as a tumor promoter in various cancers including LC [15, 16]. Activation of FGFR1 has been documented to promote epithelial-mesenchymal transition and metastasis in LC [17]. However, the mechanism by which circNINL affects LC by regulating the miR-3918/FGFR1 axis was not investigated.

In this study, we sought to investigate the influence of the circNINL/miR-3918/FGFR1 axis on the biological behavior of LC. We hypothesized that circNINL is aberrantly overexpressed in LC and may play a regulatory role in the disease's progression. Our findings aim to furnish robust data support, enhancing the understanding of the mechanistic underpinnings of LC pathogenesis.

## Materials and methods

### Patients’ consent

This study was approved by the Ethics Committee of Hainan General Hospital( Hainan Affiliated Hospital of Hainan Medical University), and written informed consent was obtained from all participants.

### Clinical specimens

Fifty-seven pairs of tumor tissues and non-tumor tissues (greater than 5 cm from tumor tissue) were collected from LC patients admitted to Hainan general hospital (Hainan affiliated hospital of Hainan medical university) from March 2017 to April 2019. Patients with primary LC who had not received chemotherapy, chemotherapy, or other treatments before surgery were included. Tissue specimens were histopathologically and clinically diagnosed and stored at − 80 °C.

### Cell culture and transfection

Human LC cell lines (H1975, A549 and H1299) and human bronchial epithelial cell line (HBE1) were cultured in Dulbecco’s Modified Eagle Medium (Gibco, NY, USA) supplemented with 10% fetal bovine serum (Gibco), 100 IU/mL penicillin (Gibco), and 100 μg/mL streptomycin (Gibco) and maintained in a humidified environment at 37 ℃ with 5% CO_2_ [18]. All cell lines were purchased from ATCC (VA, USA).

A549 cell transfection was performed using Lipofectamine 3000 (Invitrogen, CA, USA). si-circNINL#1 (ACTTTCCTGCCACCAATGATT), si-circNINL#2 (AACTTTCCTGCCACCAATGAT), si-circNINL#3 (AGAAACTTTCCTGCCACCAAT), miR-3918 mimic, miR-3918 inhibitor (in-miR-3918), NC mimic (miR-NC), and NC inhibitor (in-NC) were manufactured by GenePharma (Shanghai, China) [19], and pcDNA3.1 and pcDNA-FGFR1 vectors were designed by Invitrogen [20].

### RNase R and actinomycin D assay

RNase R resistance detection: total RNA (2 μg) was incubated with 3 U/μg RNase-R (07250, Epicentre Technologies, Mumbai, India) at 37 ℃ for 30 min and circNINL and linear NINL expression was detected by reverse transcription quantitative polymerase chain reaction (RT-qPCR).

Half-life assay: cell culture medium was added with 2 mg/mL Actinomycin D (129,935, Millipore, MA, USA) to block transcription, followed by quantitative detection of circNINL and NINL expression by RT-qPCR.

### Subcellular localization analysis

A549 cells were collected, washed with PBS, and centrifuged to discard the supernatant. Cells were subsequently resuspended in a lysis buffer, and cytoplasmic and nuclear RNAs were separated using the PARIS Kit (Invitrogen) [21]. Differential centrifugation facilitated the isolation of the cytoplasmic and nuclear components. The abundance of circNINL in the cytoplasmic and nuclear RNAs was then assessed using RT-qPCR, with U6 and 18S RNA serving as nuclear and cytoplasmic reference markers, respectively.

### RT-qPCR

Total RNA was isolated from LC tissues and cell lines using Trizol reagent (Invitrogen). Reverse transcription for CircRNA/mRNA and miRNA was separately carried out using the PrimeScript RT Reagent Kit (Takara, Tokyo, Japan) and miRNA First Strand Synthesis Kit (Takara, Japan). RT-qPCR was performed with the SYBR Green PCR Kit (Thermo Fisher Scientific, USA) on the Mx3005P QPCR System (Agilent Technologies, USA). U6 and GAPDH were selected as the internal references for miRNA and mRNA/CircRNA, respectively. Primer sequences are detailed in Table 1. The 2^−∆∆Ct^ method was applied for RNA quantification.

**Table 1:** PCR sequences

Genes	Sequences (5′–3′)
circNINL	Forward: 5′- GGAATACGAGCTCAAGTGCC-3′
	Reverse: 5′- GCCATTCTGAATCCCCTCCT-3′
miR-3918	Forward: 5′- ACACTCCAGCTGGGACAGGGCCGCAGATG -3′
	Reverse: 5′- TGGTGTCGTGGAGTCG -3′
FGFR1	Forward: 5′- TACTTCTCCGTCAATGTTTC -3′
	Reverse: 5′- GGTTTGGTGTTATCTGTTTC -3′
U6	Forward: 5′-CTCGCTTCGGCAGCACA-3′
	Reverse: 5′-AACGCTTCACGAATTTGCGT-3′
GAPDH	Forward: 5′- CACCCACTCCTCCACCTTTG -3′
	Reverse: 5′- CCACCACCCTGTTGCTGTAG -3′

circNINL, circular RNA NINL; miR-3918, microRNA-3918; FGFR1, FGF receptor 1; GAPDH, glyceraldehyde-3-phosphate dehydrogenase

### Protein expression determination

Total protein extracts were obtained by 500 μl RIPA lysis buffer (Beyotime, Shanghai, China). Protein (20 μg) was loaded onto 8% sodium dodecyl sulphate polyacrylamide gel electrophoresis (Solarbio, Beijing, China). Afterward, the sample was transferred to polyvinylidene fluoride membranes (Invitrogen) and blocked with 5% skim milk. Primary antibodies rabbit FGFR1 (ab10646, 1:10,000, Abcam), Bax (1:1000, 50,599–2-IG, Proteintech), Bcl-2 (1:1000, sc-7382, Santa Cruz Biotechnology), and GAPDH (ab9485, 1:2500, Abcam) were incubated overnight at 4℃, and horseradish peroxidase-conjugated goat anti-rabbit secondary antibody IgG (1:1000, ab181236, Abcam) was then detected for 2 h. Signals were developed by ECL kit (34,080, Thermo Fisher Scientific) and analysis was done by ImageJ software [22].

### Analysis of proliferative activity

A549 and H1299 cells after treatment were seeded in the 96-well plate at 1 × 10^4^ cells/well and supplemented with 10 μL of cell counting kit-8 reagent (Dojindo, Kumamoto, Japan) at 0, 24, 48, and 72 h for 2 h. Optical density values were recorded at 450 nm on a microplate reader (PerkinElmer Genetics, Inc., MA, USA) [23].

### Analysis of colony-forming ability

A549 and H1299 cells (100 cells/well) in 6-well plates were incubated for 14 d. After fixation with formaldehyde (Solarbio), cells were stained with 0.1% crystal violet (Solarbio). Finally, colonies were counted under a microscope (Olympus, Tokyo, Japan) [24].

### Analysis of apoptotic ability

Apoptosis of A549 and H1299 cells was examined by the Annexin V-fluorescein isothiocyanate-propidium iodide apoptosis kit (BD Biosciences, NJ, USA). Cells were collected after transfection and washed with cold PBS. The cells were then suspended with 1 × binding buffer (500 μl), mixed with 5 μl Annexin, and preserved at room temperature for 15 min away from light with V‐FITC and 5 μl PI. The percentage of apoptotic cells was assessed on an FACS Calibur flow cytometer [25].

### Examinations of invasive and migratory abilities

A549 and H1299 cells (1 × 10^5^ cells/well) were inoculated into an upper chamber pre-coated with Matrigel (5 mg/mL in cold medium, BD Transduction Laboratories, USA) containing 200 μl serum-free RPMI1-1640 (Gibco). The lower chamber was supplemented with RPMI-1640 containing 10% fetal bovine serum (500 μl). After 48 h, the invaded cells were fixed with methanol, stained with crystal violet, and counted in 5 fields.

To assess cell migration, the same experiment was performed without Matrigel [26].

### Glycolysis assay

Glucose consumption and lactate production in LC cell cultures were detected using the Glucose Assay Kit and Lactate Kit (Abcam). Relative ATP/ADP ratios were determined using the ApoSENSOR ADP/ATP Ratio Assay Kit (BioVision) [27].

### Dual luciferase reporter assay

Luciferase reporter vectors (circNINL-WT, circNINL-MUT, FGFR1 3'UTR-WT and FGFR1 3'UTR-MUT) were generated by inserting circNINL or FGFR1 3'UTR sequence containing miR-3918 target site or mutated site into pGL3 vector (Promega). The vectors were co-transfected into A549 cells with miR-3918 mimic by Lipofectamine® 3000, and after 48 h, cell luciferase activity was checked by dual luciferase detection system kit (Promega) [28].

### RNA immunoprecipitation (RIP) assay

RIP was implemented according to the procedures of Magna RIP™ Kit (Millipore, MA, USA). Cell lysates were separately incubated with Ago2 antibody and IgG antibody and added with agarose beads (Bio-Rad, CA, USA). circNINL, miR-3918 and FGFR1 expression was detected by RT-qPCR [22].

### RNA pull-down assay

miR-3918 was biotinylated to generate Bio-miR-3918 (Bio-NC as a control). A total of 2 μg cell lysate was mixed with Bio-miR-3918 (100 pmol) or Bio-NC (100 pmol). circNINL, miR-3918, and FGFR1 were subsequently determined by quantitative PCR.

### Nude mouse xenografts

Animal treatments were approved by the Animal Ethics Committee of Hainan general hospital(Hainan affiliated hospital of Hainan medical university). Ten male BALB/C nude mice (5–6 weeks old, 12 ± 4 g) were obtained from Hunan SJA Laboratory Animal Co., Ltd. (Changsha, China). Stably knocked down A549 cells and control cells (approximately 1 × 10^7^) were injected subcutaneously into the axilla of BALB/C nude mice. Width and length were measured with calipers from day 3 after transplantation, and tumor growth was monitored weekly, and tumor volume was calculated: (Width^2^ × length)/2. After 4 weeks, mice were euthanized and tumor weights were measured. Tumors were assayed for Ki-67 and FGFR1 expression using immunohistochemistry as previously described using antibodies Ki-67 (ab16667, 1:100, Abcam) and FGFR1 (ab10646, 1:1500, Abcam) [29].

### Statistical analysis

SPSS 21.0 (IBM, NY, USA) was of utility to analyze the data. Kolmogorov–Smirnov test showed that the data were normally distributed, and the results were expressed as mean ± standard deviation. Comparisons between two groups were performed using t test, and comparisons among multiple groups were performed using One/Two-Way one-way analysis of variance. Chi-square test was used to analyze the direct correlation between circNINL and the clinicopathology of LC patients, Pearson correlation analysis to evaluate the correlation between circNINL/miR-3918/FGFR1, and Kaplan–Meier analysis to assess patient survival. * *P* < 0.05 indicates a significant difference.

## Results

### High expression of circNINL is associated with poor prognosis in LC patients

**Fig. 1:**
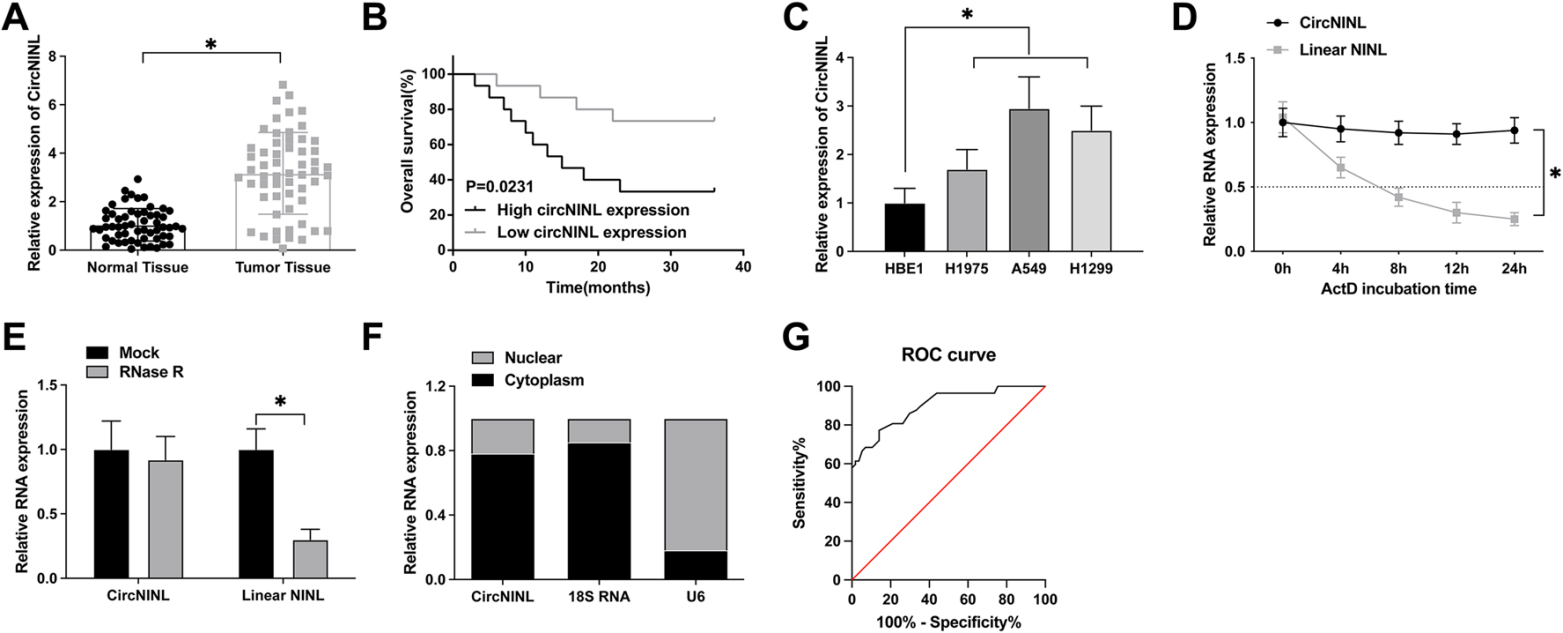
Elevated expression of circNINL correlates with poor prognosis in LC patients. **A** RT-qPCR assessment of circNINL levels in normal and tumor tissues. **B** Kaplan–Meier survival analysis illustrating prognostic outcomes in LC patients. **C** RT-qPCR quantification of circNINL in various LC cell lines. **D** RNase R resistance assay to determine the stability of circNINL. **E** Act D assay used to ascertain the half-life of circNINL. **F** Nuclear-cytoplasmic fractionation experiments revealing the subcellular localization of circNINL in A549 cells. **G** ROC curve analysis evaluating the clinical diagnostic value of circNINL in LC. Data are presented as mean ± SD (n = 3). *P < 0.05 indicates statistical significance

In a focused analysis encompassing 57 LC patients, this study delved into the differential expression of circNINL in LC versus adjacent normal tissues, revealing a notable upregulation in the tumor specimens (Fig. 1A). This finding aligns with an emerging pattern linking circNINL levels to key clinical-pathological features such as tumor size, lymph node metastasis, and TNM staging (Table 2), though its expression showed no significant correlation with other clinical metrics. Notably, patients exhibiting higher circNINL levels corresponded with poorer overall survival (Fig. 1B), underscoring its prognostic significance. When assessing circNINL expression across different cell lines, including human LC cell lines (H1975, A549, and H1299) and a human bronchial epithelial cell line (HBE1), we observed a striking overexpression in the cancer cell lines, especially A549 (Fig. 1C).

**Table 2:** Correlation between clinicopathological features and expression levels of circNINL in patients with lung cancer

Characteristic	Cases	circNINL expression		*P*
	*n* = 57	High (*n* = 28)	Low (*n* = 29)	
Age(year)				0.8889
≥ 60	27	13	14	
< 60	30	15	15	
Gender				0.3489
Male	38	17	21	
Female	19	11	8	
Smoking				0.6813
Yes	31	16	15	
No	26	12	14	
Tumor size (cm)				< 0.0001*
≥ 5	21	18	3	
< 5	36	10	26	
TNM stage				0.0102*
I–II	24	7	17	
II–IV	33	21	12	
Histological type				0.6230
Squamous	14	7	7	
Adenocarcinoma	24	13	11	
Large cell lung cancer	19	8	11	
Lymph node metastasis				0.0054*
Negative	31	10	21	
Positive	26	18	8	

Chi-square test was used to analyze the correlation between circNINL and clinicopathological characteristics of lung cancer patients

This overexpression prompted further investigation into circNINL's molecular function and stability, where RNase R and Actinomycin D assays validated its circular nature and enhanced stability compared to linear NINL mRNA (Fig. 1D, E). Intriguingly, circNINL was predominantly localized in the cytoplasm of LC cells (Fig. 1F), suggesting a role in miRNA sequestration and post-transcriptional gene regulation. The diagnostic potential of circNINL, evidenced by a ROC curve with an area under the curve of 0.8981 (95% confidence interval: 0.8428 to 0.9535, Fig. 1G), highlights its relevance as an oncogenic biomarker and a critical player in LC progression.

### Effects of circNINL knockdown on suppressing LC cell activities and glycolysis

**Fig. 2:**
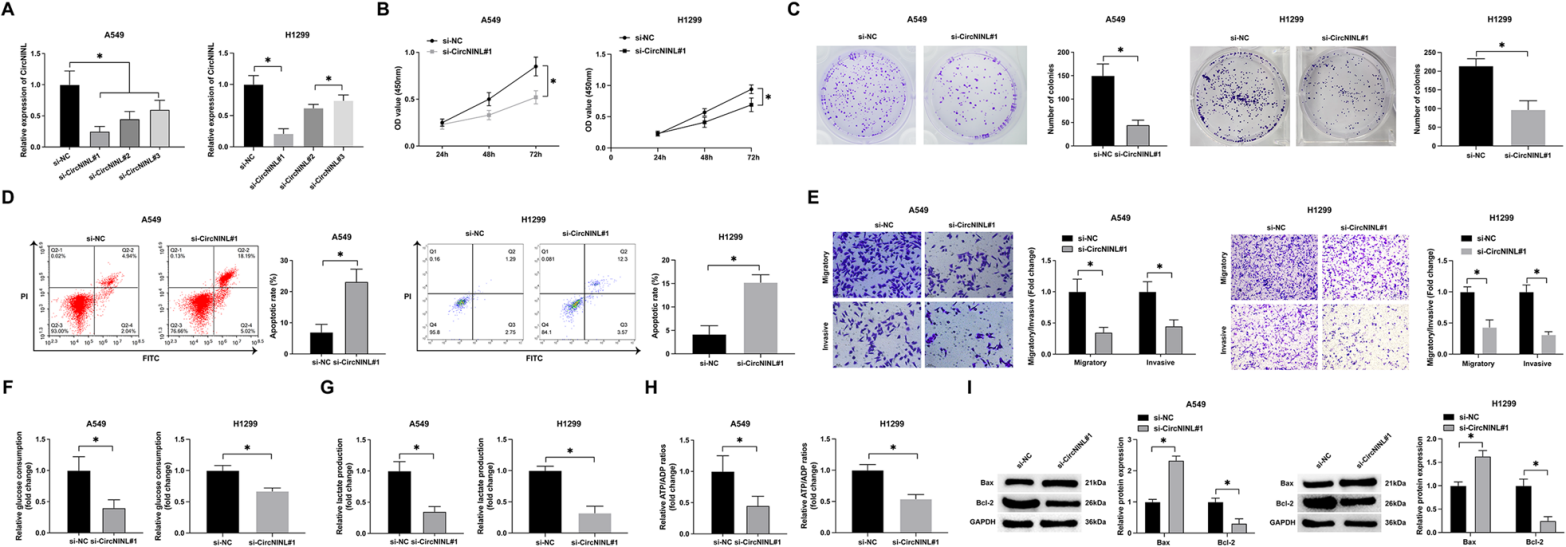
Knockdown of circNINL inhibits proliferation, migration, invasion, and glycolysis in A549 and H1299 cells, and induces apoptosis. sh-circNINL was transfected into A549 and H1299 cells. **A** RT-qPCR analysis of the transfection efficiency of si-circNINL#1#2#3. **B** CCK-8 assay to evaluate cell proliferation activity. **C** Colony formation assay assessing cell proliferative capacity. **D** Flow cytometry to measure cell apoptosis. **G** Transwell assay determining cell migration and invasion capabilities. **F**–**H** Glucose consumption, lactate production, and ATP/ADP ratios were measured using corresponding assay kits. **I** Western blot analysis was employed to assess the protein expression levels of Bax and Bcl-2 in the cells. Data are presented as mean ± SD (*n* = 3). *P < 0.05 indicates statistical significance

In our pursuit to elucidate the influence of circNINL on the biological functions of LC cells, we transfected si-circNINL into A549 and H1299 cells, attaining a transfection efficiency of approximately 82%. Strikingly, si-circNINL#1, #2, and #3 significantly downregulated circNINL expression, as shown in Fig. 2A. Given the superior inhibitory efficacy of si-circNINL#1, it was utilized for all subsequent functional loss experiments. Proliferation assessments, encompassing both CCK-8 and colony formation assays, indicated a marked reduction in cellular proliferation and colony formation following circNINL knockdown (Fig. 2B, C). Flow cytometric analysis revealed that silencing circNINL markedly induced apoptosis in these cells (Fig. 2D). Furthermore, Transwell assays demonstrated a significant decline in the migratory and invasive capabilities of the cells post circNINL depletion (Fig. 2E).

Considering the predominant reliance of aggressively growing malignant cells on glycolysis for energy, the effects of circNINL on this metabolic pathway were also investigated. Knockdown of circNINL led to a notable decrease in glucose consumption, lactate production, and the ATP/ADP ratio in the cancer cells (Fig. 2F–H). Additionally, knockdown of circNINL markedly upregulated the protein expression of Bax while concomitantly diminishing that of Bcl-2 in the cells (Fig. 2I). These results collectively delineate that the reduction of circNINL curbs LC cell proliferation, migration, invasion, and glycolytic activity while inducing apoptosis, thus underscoring its potential role as a key target in LC therapeutics.

### circNINL targets miR-3918 expression

**Fig. 3:**
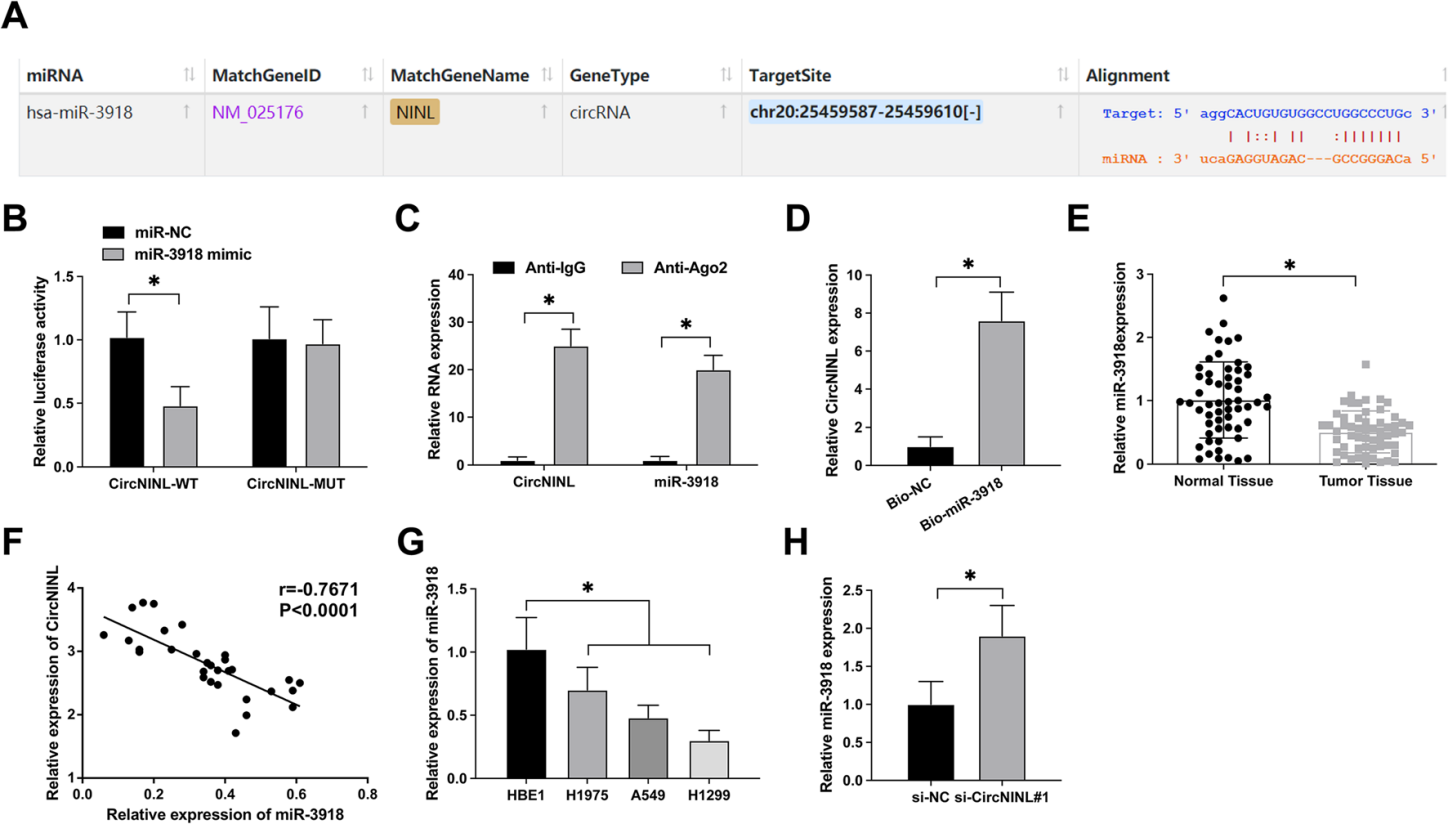
circNINL functioning as a ceRNA for miR-3918. **A** Bioinformatic prediction at starbase.sysu.edu.cn identifies potential interaction sites between circNINL and miR-3918. **B**–**D** Evaluative assays, encompassing dual-luciferase reporter, RIP, and RNA-pull down tests, were conducted to delineate the interactive dynamics between circNINL and miR-3918. **E** Expression of miR-3918 across LC and normal tissue samples assessed via RT-qPCR. **F** Pearson correlation analysis was utilized to decipher the associative degrees between miR-3918 and circNINL expressions. **G** RT-qPCR quantification of miR-3918 in various LC cell lines vis-à-vis normal cellular counterparts. **H** Investigation of miR-3918 expression post the silencing of circNINL, conducted through RT-qPCR. The collected data are expressed as mean ± SD for three replicates (*n* = 3), where *P < 0.05 indicates statistical significance

To elucidate the ceRNA mechanism of circNINL, we engaged the bioinformatics platform https://starbase.sysu.edu.cn for potential miRNA interactions. Our analysis highlighted miR-3918 as a key miRNA exhibiting prospective binding sites with circNINL (Fig. 3A). To experimentally validate this interaction, we performed a dual-luciferase reporter assay. Co-transfection with circNINL-WT and miR-3918 mimic significantly attenuated luciferase activity, illustrating a functional interaction (Fig. 3B). This finding was further bolstered by RIP assays, which demonstrated a robust enrichment of both circNINL and miR-3918 in Ago2 immunocomplexes (Fig. 3C). An RNA-pulldown assay using a bio-miR-3918 probe successfully precipitated circNINL, reinforcing their specific interaction (Fig. 3D). Clinically, a marked downregulation of miR-3918 in LC tissues relative to adjacent normal tissues was observed, as demonstrated in Fig. 3E. Clinical correlation studies further revealed a significant inverse relationship between miR-3918 and circNINL levels (Fig. 3F). Comparatively, miR-3918 was found to be substantially depleted in human LC cell lines versus normal bronchial epithelial HBE1 cells (Fig. 3G). Strikingly, circNINL knockdown in A549 cells markedly increased miR-3918 expression (Fig. 3H), suggesting a regulatory axis wherein circNINL modulates miR-3918 availability. This discovery offers new insights into the intricate molecular interplay governing LC pathogenesis and implicates circNINL as a pivotal regulator in the miRNA-mediated oncogenic landscape.

### miR-3918 knockdown attenuates si-circNINL-mediated effects on LC cells

**Fig. 4:**
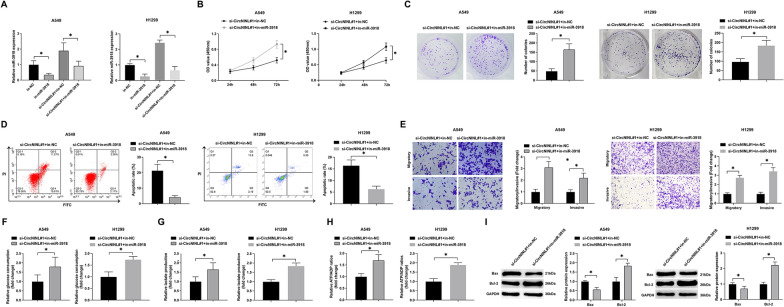
Counteraction of circNINL knockdown's inhibitory impact on LC cellular aggressiveness through miR-3918 inhibition. In a bid to elucidate the interactive dynamics, A549 and H1299 cells underwent co-transfection with si-circNINL and a miR-3918 specific inhibitor. **A** Efficiency of the si-circNINL and miR-3918 inhibitor transfections were quantitatively corroborated via RT-qPCR. **B** CCK-8 assay was systematically employed to gauge cellular proliferation. **C** Colony formation assay provided insight into the proliferative robustness of the cells. **D** Apoptotic frequencies were meticulously quantified through flow cytometry. **E** Transwell assays were meticulously executed to map out the nuances of cellular migration and invasion. **F**–**H** Key metabolic indicators, including glucose uptake, lactate secretion, and the ATP/ADP ratio, were comprehensively quantified employing specialized assay kits. **I** Western blot analysis was employed to assess the protein expression levels of Bax and Bcl-2 in the cells. Data, presented as mean ± SD, stem from triple independent repetitions (*n* = 3). An asterisk (*P < 0.05) flags statistical significance, underscoring the robustness of the findings

To probe the role of miR-3918 in circNINL-mediated regulation of LC progression, functional rescue experiments were conducted in A549 and H1299 cell lines. We co-transfected these cells with in-miR-3918 and si-circNINL, noting that while the inhibitor did not affect miR-3918 RNA expression, circNINL knockdown led to an upsurge in miR-3918 levels (Fig. 4A). Proliferation assays, including CCK-8 and colony formation tests, revealed that circNINL knockdown significantly curtailed cellular proliferation rates and numbers, effects which were mitigated by the concurrent knockdown of miR-3918 (Fig. 4B, C). Flow cytometric analysis indicated a marked enhancement in apoptosis in circNINL-silenced cells, a trend reversed by miR-3918 inhibition (Fig. 4D). Moreover, the suppressive impact of circNINL knockdown on cell invasion and migration was effectively counterbalanced by miR-3918 knockdown (Fig. 4E). Notably, reductions in glucose consumption, lactate production, and the ATP/ADP ratio following circNINL knockdown were also nullified by miR-3918 inhibition (Fig. 4F–H). Western blot analysis revealed that silencing circNINL significantly upregulated Bax protein levels and downregulated Bcl-2 expression in cells. Notably, this protein expression alteration was hindered by knockdown of miR-3918 (Fig. 4I). These findings collectively illuminate circNINL's crucial involvement in driving the biological behaviors and aerobic glycolysis of LC cells, predominantly through targeting and modulating miR-3918.

### FGFR1 is a functional target of miR-3918

**Fig. 5:**
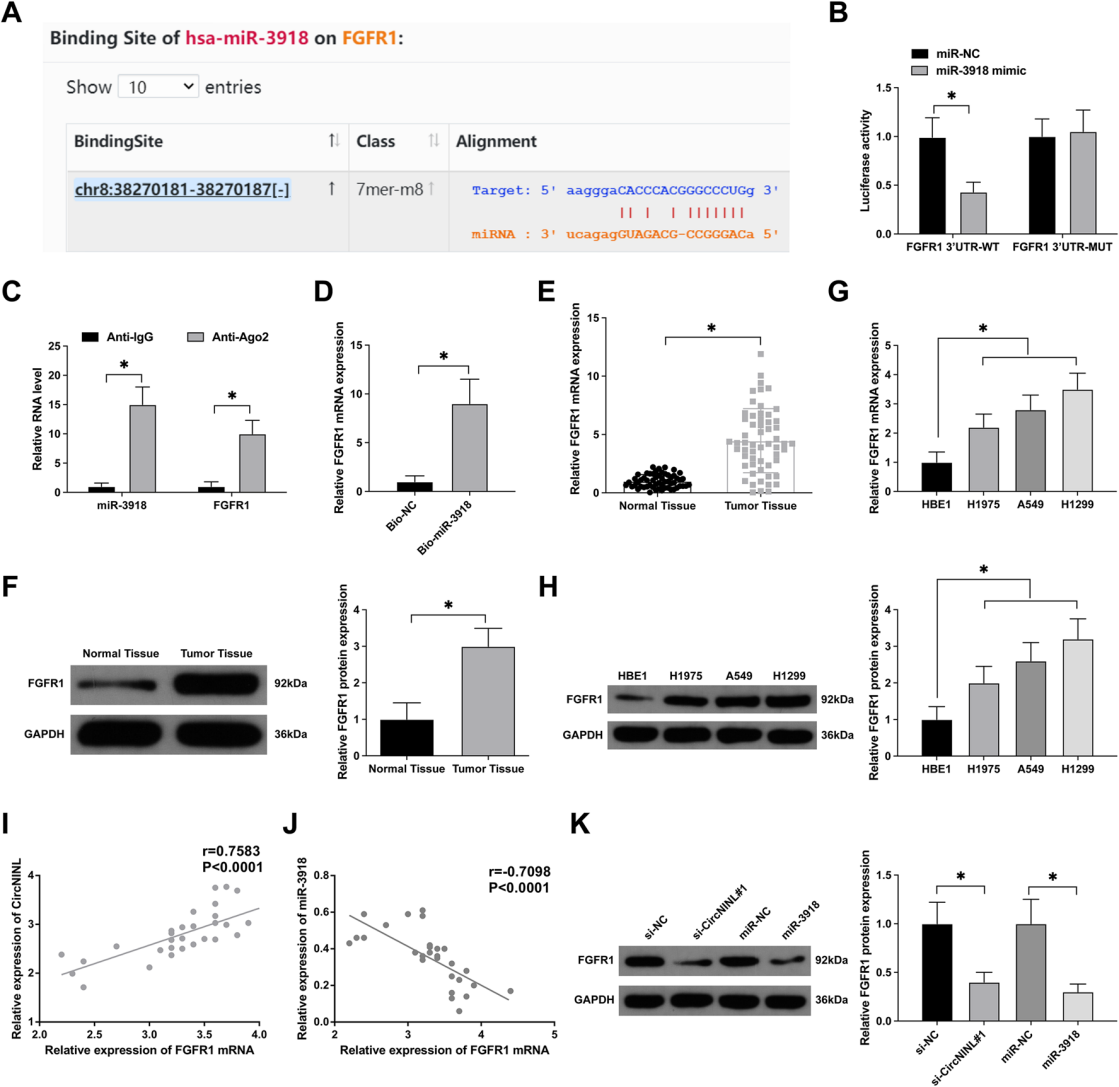
FGFR1 is a functional target of miR-3918 in LC cells. **A** Bioinformatics predictions of binding sites between FGFR1 and miR-3918 were made using the website https://starbase.sysu.edu.cn. **B**–**D** Interactions between FGFR1 and miR-3918 were probed via dual-luciferase reporter assays, RIP, and RNA-pull down experiments. **E**–**H** RT-qPCR and Western blot analyses evaluated FGFR1 expression in LC tissues and cells relative to normal tissues. **I**, **J** Pearson correlation analysis assessed the associations among FGFR1, miR-3918, and circNINL. **K** Western blot analysis explored FGFR1 expression post downregulation of circNINL or upregulation of miR-3918. Data are expressed as mean ± SD (*n* = 3). A *P < 0.05 denotes statistical significance

Subsequent to our initial discoveries, further exploration into the downstream mRNA targets of miR-3918 was undertaken using bioinformatics analysis (https://starbase.sysu.edu.cn), revealing a potential binding affinity between miR-3918 and FGFR1 (Fig. 5A). A significant reduction in luciferase activity was observed upon co-transfection of miR-3918 mimic and FGFR1-WT, as evidenced by a dual-luciferase reporter assay (Fig. 5B). Additionally, RIP assays indicated substantial enrichment of miR-3918 and FGFR1 in the Anti-Ago2 group (Fig. 5C). Comparative enrichment of FGFR1 was also observed in the Bio-miR-3918 probe group versus the Bio-NC group, as demonstrated in RNA-pull down assays (Fig. 5D).

Concomitantly, analyses of LC tissues revealed a significant elevation in FGFR1 mRNA and protein levels compared to normal tissues (Fig. 5E, F). This upregulation was mirrored in LC cell lines, where FGFR1 expression surpassed that of normal cells (Fig. 5G, H). Moreover, clinical correlation analyses suggested a positive correlation between FGFR1 and circNINL expressions, and a negative correlation with miR-3918 (Fig. 5I, J). Western blot assays further corroborated these findings, showing that downregulation of circNINL or overexpression of miR-3918 led to decreased FGFR1 expression in A549 cells (Fig. 5K). Collectively, these data robustly nominate FGFR1 as a downstream target gene of miR-3918, underlining its potential role in the molecular circuitry modulating LC progression.

### FGFR1 overexpression reverses si-circNINL-mediated effects on LC cells

**Fig. 6:**
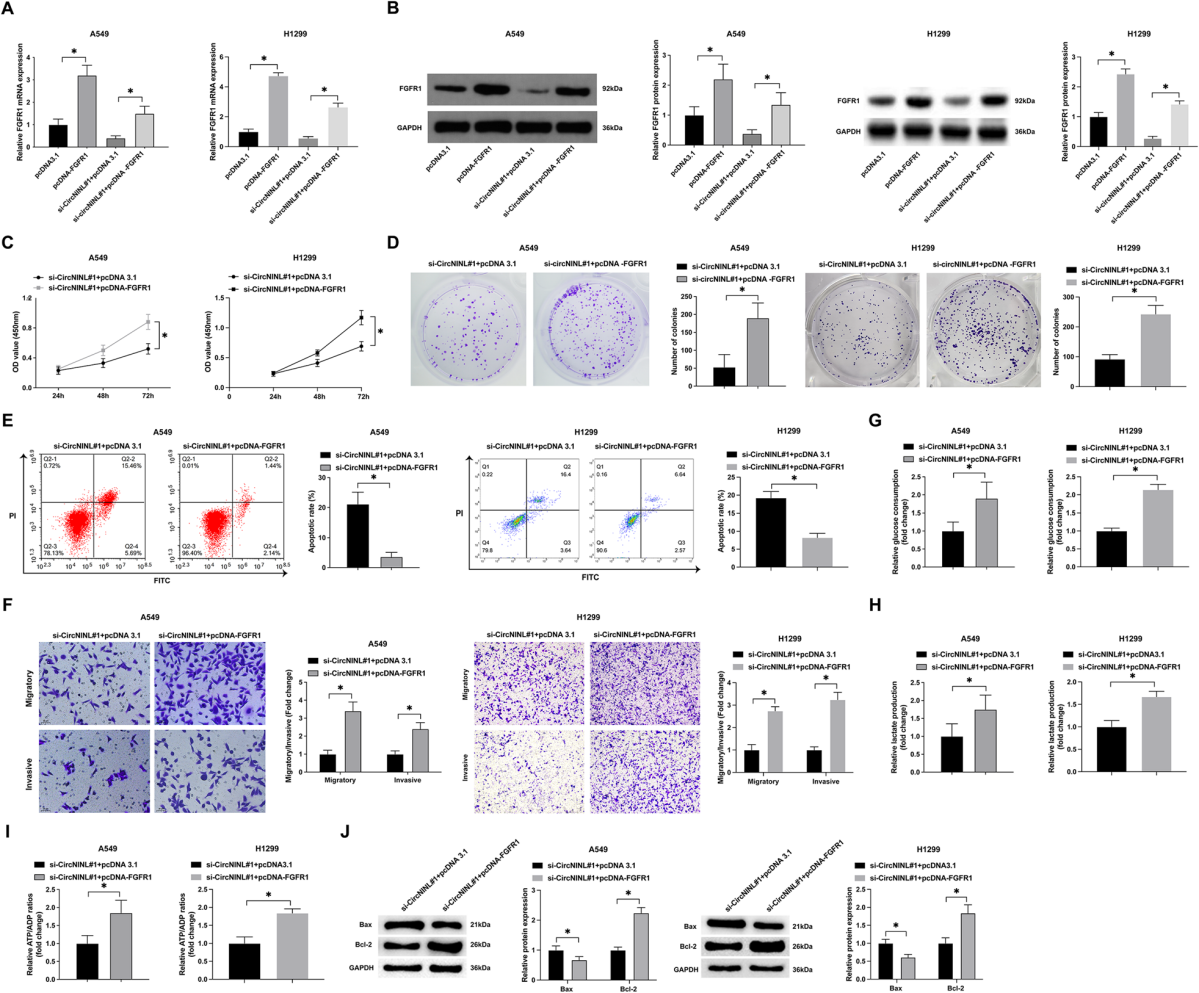
Counteractive effect of FGFR1 overexpression on circNINL knockdown-driven LC aggressiveness. In this experiment, si-circNINL and pcDNA 3.1-FGFR1 were co-transfected into A549 and H1299 cells. **A**, **B** RT-qPCR and Western blot analyses were employed to measure FGFR1 expression in A549 and H1299 cells, thereby providing a molecular readout of the overexpression efficiency. **C** CCK-8 assay offered insights into alterations in cell proliferation rates post-transfection, serving as a benchmark for functional impact. **D** The ability of cells to proliferate was further scrutinized using colony formation assays, offering a complementary perspective on cellular growth dynamics. **E** Apoptotic indices were meticulously quantified through flow cytometry, shedding light on the cellular response to genetic manipulation. **F** Transwell assays afforded a quantitative evaluation of the migratory and invasive capabilities of the cells, highlighting the impact of circNINL and FGFR1 modulation on these critical aspects of cancer pathology. **G**–**I** Metabolic shifts, encompassing glucose consumption, lactate production, and the ATP/ADP ratio, were precisely quantified using specific assay kits. **J** Western blot analysis was employed to assess the protein expression levels of Bax and Bcl-2 in the cells. Data are articulated as mean ± SD (*n* = 3). Significance was determined at *P < 0.05

To elucidate the role of FGFR1 within the circNINL-regulated oncogenic trajectory in LC, we executed an array of functional rescue assays. A549 and H1299 cells, when subjected to simultaneous transfection with pcDNA 3.1-FGFR1 and si-circNINL, exhibited a pronounced reversal of si-circNINL's suppressive influence on FGFR1 expression, as illustrated in Fig. 6A, B. Investigations via CCK-8 and colony formation assays revealed that the knockdown of circNINL substantially curtailed the proliferation rate and number of cells. This attenuation, however, was effectively abrogated by FGFR1 overexpression (Fig. 6C, D). Flow cytometric analysis further indicated that circNINL knockdown significantly elevated apoptotic rates, an effect mitigated by the overexpression of FGFR1 (Fig. 6E).

In terms of cellular dynamics, the repressive effect of circNINL knockdown on invasion and migration capabilities was conspicuously reversed upon FGFR1 overexpression (Fig. 6F). Moreover, the metabolic profiling showed that the reduction in glucose uptake, lactate secretion, and the ATP/ADP ratio, consequent to circNINL downregulation, was counterbalanced by overexpressed FGFR1 (Figs. 6G–I). Western blot assays indicated that the modulatory effects of circNINL knockdown on Bax and Bcl-2 protein levels were reversed by the overexpression of FGFR1 (Fig. 6J). These findings collectively highlight that circNINL, by intricately modulating the miR-3918/FGFR1 axis, orchestrates a multifaceted regulatory network that propels the malignant and metabolic phenotype of LC cells, thereby underscoring a nuanced mechanistic paradigm in the pathogenesis of LC.

### Knockdown of circNINL inhibits LC tumor growth in vivo

**Fig. 7:**
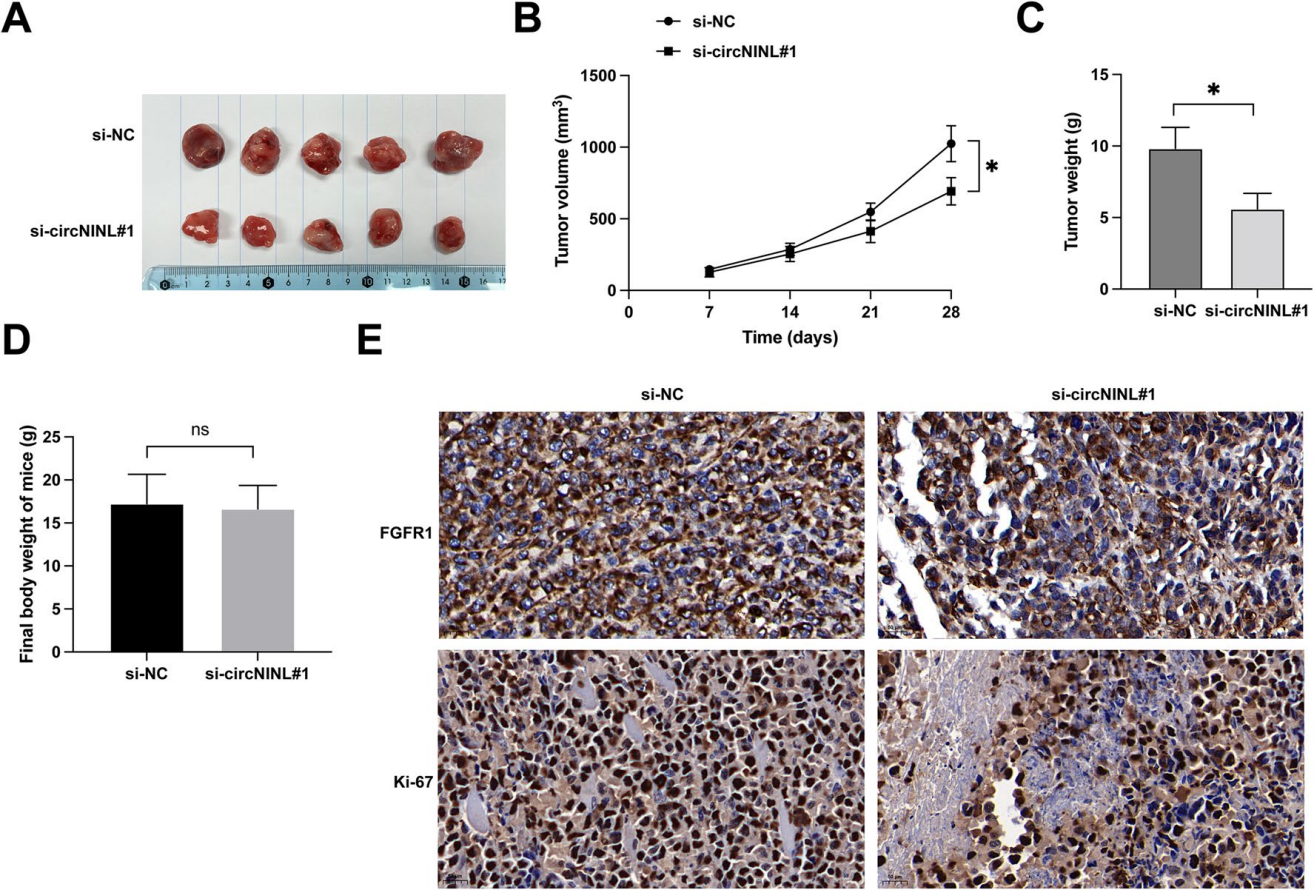
circNINL knockdown suppresses in vivo tumor growth in LC. **A**–**C** Depictions of the experimental outcomes include representative images of the tumors, alongside analyses of tumor volume and weight, offering a direct measure of the in vivo growth dynamics following circNINL knockdown. **D** Final body weight of nude mice. **E** IHC staining was meticulously applied to ascertain the expression levels of Ki-67 and FGFR1 proteins within the tumor tissues. Data are articulated as mean ± SD (*n* = 5). Significance was determined at *P < 0.05

To substantiate our in vitro observations, rigorous in vivo assessments were implemented. Nude mice were subcutaneously inoculated with A549 cells in which circNINL had been stably silenced. Figures 7A–C illustrates a notable suppression in both the growth velocity and tumor mass in the circNINL-depleted group. Notably, the knockdown of circNINL exerted no discernible effect on the overall body weight of the hosts (Fig. 7D), implicating that the modulations in tumor growth were specifically ascribed to the targeted downregulation of circNINL, rather than any alterations in general health or nutritional condition of the mice. Moreover, a consequent attenuation in the expression levels of Ki-67 and FGFR1 was observed following circNINL knockdown (Fig. 7E). These in vivo findings compellingly endorse the proposition that circNINL plays a crucial role in augmenting LC tumor proliferation, thereby underscoring its vital influence within the oncogenic landscape.

## Discussion

LC is currently the most frequently diagnosed cancer [30]. Although significant progress has been made in the treatment of LC, the 5-year survival rate and prognosis of LC patients are still poor [31, 32]. CircRNAs have been considered potentially attractive human cancer markers due to their special structure and wide distribution [33]. In this study, we discovered that circNINL affected the cellular biological behavior of LC and specified that circNINL bound to miR-3918 to regulate FGFR1 expression and enhance malignancy and glycolysis of LC cells.

CircRNAs, mainly located in the cytoplasm, are not affected by RNA exonuclease and are not easily degraded, having potential values for prognosis/diagnosis of diseases [34]. CircPTCH1 and circSETD3 have tumor-enhancing effects in LC and can be used as a novel potential therapeutic and diagnostic biomarker for LC [35, 36]. Likewise, we found that circNINL was highly expressed in LC and abundantly expressed in the cytoplasm. Additionally, we ensured that high circNINL expression predicted poor prognosis in LC patients. It has been reported that circNINL is highly expressed in breast cancer, and knocking down its expression can inhibit the malignant behaviors of tumor cells [12]. This study confirmed that circNINL also has a similar role in LC. By knocking down circNINL expression in LC cells, the malignant activities of LC cells were blocked. Abnormal proliferation, migration, and invasion in cancer cells are attributed to aerobic glycolysis, and controlling aerobic glycolysis is also a therapeutic target for cancer management [4]. Therefore, we performed related experiments and found that circNINL knockdown inhibited glycolysis by reducing glucose consumption, lactate production and ATP/ADP ratio, further supporting that circNINL exerts a therapeutic effect by inhibiting its expression in LC.

CircRNAs have been shown to act as ceRNAs to counteract miRNAs, thereby regulating cancer progression. Therefore, we predicted circNINL-related miRNAs by bioinformatics analysis and demonstrated that miR-3918 acted as a downstream miRNA of circNINL in LC. Accumulating evidence suggests that miRNAs act as oncogenes or tumor suppressor genes in various cancers, including LC [37]. miR-3918 has been reported to act as a tumor suppressor in gastric cancer [10] and hepatocellular carcinoma [14]. Similarly, we determined that downregulating miR-3918 rescued the inhibition of cell growth and aerobic glycolysis in LC cells by knocking down circNINL.

In the present study, we found that miR-3918 directly targeted the 3'UTR of FGFR1 in LC, leading to the degradation of FGFR1 and repressing its transcription. FGFR1 functions as a tumor promoter in various cancers including LC, non-small cell LC, and lung adenocarcinoma [38]. FGFR1 is frequently focally amplified in LC cell lines [39], mainly because these cells depend on FGFR1 activity for cell growth. Furthermore, inhibition of FGFR1 has been reported to inhibit cell growth [40]. In a previous study, miR-198-mediated suppression of FGFR1 is attributable to the blockade of LC cell proliferation [41]. Here, we checked the increase in FGFR1 expression in LC, and confirmed that circNINL promoted carcinogenesis and aerobic glycolysis by increasing FGFR1 expression in LC.

Although this study confirmed the role of circNINL/miR-3918/FGFR1 in LC cell lines, there is a lack of sufficient clinical experiments to validate the impact of the circNINL/miR-3918/FGFR1 axis on LC therapy. In addition, the drug resistance of LC has become difficult in the treatment of LC, and it is necessary to explore the effect of the circNINL/miR-3918/FGFR1 axis on the drug resistance of LC cell lines in subsequent studies.

## Conclusion

Shortly, circNINL promotes carcinogenesis and aerobic glycolysis by regulating the miR-3918/FGFR1 axis in LC. This discovery may provide new ideas for new target therapy and diagnosis of LC. However, this study also has some limitations: circNINL has not been detected in LC clinical serum samples; multi-center trials and animal experiments are needed.

## Data Availability

The data are available from the corresponding author upon request.
